# Assessing fluid volume and determining outcomes of acute heart failure using plasma human atrial natriuretic peptide

**DOI:** 10.1007/s10157-023-02333-1

**Published:** 2023-03-20

**Authors:** Yuya Suzuki, Tadashi Otsuka, Yuki Yoshioka, Tomomichi Iida, Shingo Maruyama, Hirofumi Watanabe, Ryohei Kaseda, Suguru Yamamoto, Yoshikatsu Kaneko, Shin Goto, Ryuji Aoyagi, Ichiei Narita

**Affiliations:** 1 Division of Clinical Nephrology and Rheumatology, Kidney Research Center, Niigata University Graduate School of Medical and Dental Sciences, 1-757 Asahimachi, Chuo-Ku, Niigata, 951-8510 Japan; 2 Division of Nephrology and Hypertension, Department of Internal Medicine, The Jikei University Daisan Hospital, Tokyo, Japan; 3 Department of Nephrology, Tachikawa General Hospital, Niigata, Japan

**Keywords:** Hemodialysis, Human atrial natriuretic peptide, Mortality, Acute heart failure, Cardiovascular disease

## Abstract

**Background:**

The post-dialysis plasma level of human atrial natriuretic peptide (hANP) reflects the fluid volume in patients on hemodialysis. The threshold hANP level is reportedly 100 pg/mL; however, the clinical usefulness of the threshold hANP level for volume control has not been sufficiently studied.

**Methods:**

We conducted a single-center, retrospective, observational study that included 156 hemodialysis patients without atrial fibrillation. First, we examined the usefulness of the threshold hANP level (100 pg/mL) for predicting hypoxemia due to congestion in a short-term observational study from December 30, 2015 to January 5, 2016. Subsequently, we conducted a 5-year follow-up study wherein the outcomes were hospitalization due to acute heart failure (AHF), development of cardiovascular diseases (CVD), and all-cause death. Finally, we collected echocardiography data to investigate the relationship between cardiac function and hANP.

**Results:**

Our short-term observational study showed that patients with an hANP level ≥ 100 pg/mL developed hypoxemia due to congestion (odds ratio, 3.52; 95% confidence interval, 1.06–11.71; P = 0.040). At the 5-year follow-up, patients with an hANP level ≥ 100 pg/mL had significantly higher rates of hospitalization due to AHF, CVD, and all-cause death based on the log-rank test (P = 0.003, P = 0.019, P < 0.001, respectively). Cardiac disfunctions were significantly associated with the high hANP level.

**Conclusions:**

The hANP level is indicative of both fluid volume and cardiac dysfunction. A threshold hANP level of 100 pg/mL can serve as a predictive marker for AHF and a practical indicator for volume control.

**Supplementary Information:**

The online version contains supplementary material available at 10.1007/s10157-023-02333-1.

## Introduction

Volume overload leads to the development of heart failure (HF), which contributes to the high mortality in patients on hemodialysis [1, 2]. To optimize fluid volume evaluation, patients’ appropriate dry weight (DW) needs to be set. Various markers to assess fluid volume, such as blood pressure (BP), edema, cardiothoracic ratio (CTR), bioimpedance analysis, and natriuretic peptides, have been used [3]. However, in some cases, these markers may not accurately reflect the actual fluid volume. Therefore, volume overload is still common in patients on hemodialysis, and defining the appropriate fluid volume remains a challenge.

Human atrial natriuretic peptide (hANP) is a hormone synthesized mainly in the left atrium in response to atrial wall stress and volume overload [4]. Unlike other natriuretic peptides, such as B-type natriuretic peptide (BNP) or N-terminal pro BNP (NT-pro BNP), the half-life of hANP is short (2–3 min); therefore, the hANP level more accurately reflects the fluid volume at the time of measurement [5]. For this reason, the hANP level after a hemodialysis session is considered a useful marker when adjusting DW [6]. Preventing congestive HF is an important short-term goal of adjusting DW in clinical practice. Although several studies showed the relationship between the hANP level and long-term clinical outcomes such as mortality and CVD [7–10], there have been no studies that showed the high level of hANP was related to the development of congestive HF in a short-term observation. We consider that the usefulness of hANP for adjusting DW should also be evaluated by a short-term clinical outcome because the hANP level reflected temporary volume status at the time of measuring. In addition, the appropriate threshold hANP level has not been studied enough. Although only the guidelines of the Japanese Society for Dialysis Therapy showed that the threshold hANP level is 50–100 pg/mL when DW is achieved [11], no studies had evaluated the usefulness of the threshold in clinical outcomes, such as congestive HF. Furthermore, the extent to which cardiac dysfunction impacts the plasma levels of hANP remains uncertain.

Based on the above, we sought to clarify the usefulness of the hANP threshold level for adjusting DW. First, we examined whether patients on hemodialysis with an hANP level ≥ 100 pg/mL tended to develop hypoxemia due to congestion in a short-term observational study. Subsequently, we conducted a 5-year clinical follow-up to compare the rates of hospitalization due to acute HF (AHF), cardiovascular disease (CVD), and all-cause death between patients with an hANP level ≥ 100 pg/mL and < 100 pg/mL. In addition, we investigated the impact of cardiac function on the hANP level to evaluate the usefulness of hANP in patients with cardiac dysfunction.

## Materials and methods

### Study protocol

This study was a single-center, retrospective, observational study conducted at the Tachikawa General Hospital. We initially included 175 patients who underwent hemodialysis from December 30, 2015 to January 5, 2016. As patients with atrial fibrillation (AF) were reported to have high plasma hANP levels [12], 18 patients with persistent or paroxysmal AF were excluded. In addition, one patient was excluded because of disapproval. Finally, 156 patients were included in the study. All the included patients are outpatients and none of them admitted to the hospital during the above period. This study was approved by the Institutional Review Board of Tachikawa General Hospital (# 12000056) and was performed in accordance with the Declaration of Helsinki. All patients provided written informed consent.

### Short-term observational study

During three consecutive dialysis sessions from December 30, 2015 to January 5, 2016, clinical data were collected by reviewing patient medical records. Blood tests, including measurement of hANP levels, were performed immediately after the third session. DW of the patients did not change during the three consecutive dialysis sessions. The clinical outcome was the occurrence of hypoxemia due to congestion, which was defined as pre-dialysis low peripheral oxygen saturation (SpO_2_) level (< 96%), which improved with ultrafiltration to more than 96%. All of the patients exhibited more than 96% SpO2 after the dialysis session and there were no instances of persistent low SpO2 levels in this study.

### Clinical follow-up

From January 5, 2016 to January 5, 2021, clinical data were retrospectively collected from the medical records of the subjects. During the follow-up period, we regularly adjusted DW of the patients according to the fluid volume indicators, such as CTR, edema, and BP. The primary outcome was hospitalization due to AHF, and the secondary outcomes were development of CVD or all-cause death. AHF was defined as low SpO_2_ level (< 90%) that improved with ultrafiltration. CVD included acute coronary syndrome, cerebral infarction, cerebral hemorrhage, subarachnoid hemorrhage, aortic dissection, and ruptured aortic aneurysm. To evaluate the effectiveness of the threshold hANP level in elderly patients, we extracted data from patients aged ≥ 75 years. The age of elderly people was defined according to the proposal from the Joint Committee of Japan Gerontological Society and the Japan Geriatrics Society [13].

### Echocardiography

The most recent echocardiography data before or after measuring hANP level were collected by reviewing patient medical records. The examinations were performed using Xario SSA-660A (TOSHIBA, Japan). Left ventricular ejection fraction (LVEF) was calculated by the modified Simpson’s method. Systolic dysfunction was defined as LVEF < 50% according to the European Society of Cardiology (ESC) Guidelines for Acute and Chronic Heart Failure [14]. Diastolic function was classified into three groups (normal, indeterminate, diastolic dysfunction) in accordance with the guideline of American Society of Echocardiography and the European Association of Cardiovascular Imaging [15]. In patients with LVEF ≥ 50%, those with diastolic dysfunction were classified as heart failure with preserved ejection fraction (HFpEF) [15]. Those without systolic or diastolic dysfunction were classified as having normal cardiac function.

### Statistical analysis

All normally distributed data were presented as mean ± standard deviation, whereas non-normally distributed data were presented as median (interquartile range). The differences between the two groups were compared by chi-squared tests or Fisher's exact tests for proportions, unpaired Student or Welch t-tests for continuous variables with normal distribution, and the Wilcoxon signed-rank test for continuous variables with non-normal distribution. The distinctions among the three groups were evaluated through chi-squared tests or Fisher's exact tests for proportions, one-way ANOVA for continuous variables with normal distribution, and the Kruskal–Wallis test for continuous variables with non-normal distribution. The odds ratios of the two groups were calculated by the cross-tabulation table and presented with 95% confidence intervals (CIs). Event-free survival curves were estimated with the Kaplan–Meier method, and estimates were compared using the log-rank test. Patients lost to follow-up were censored at the date of the last clinical record. The threshold hANP level in predicting AHF, CVD and all-cause death was analyzed by utilizing Receiver Operating Characteristics (ROC) curve analysis. Cox proportional hazard model analysis was conducted to evaluate the independent relationship between hANP and outcomes. In multivariable models, we selected five variables related to the development of AHF (sex, age, hemoglobin, albumin, and history of diabetes) based on a previous report [16]. In the analysis of echocardiography data, we performed a Cox proportional hazard model analysis to evaluate the independent relationship between hANP and cardiac functions, after excluding two patients without echocardiography data. The results were presented as hazard ratios with 95% CIs. All reported P-values were two-sided, and values of < 0.05 were considered statistically significant. All statistical analyses were conducted using JMP version 16.1.0 (SAS Institute, Cary, NC, USA) or GraphPad Prism ver.9.2.0 for Windows (GraphPad Software, San Diego California USA).

## Results

### Short-term observational study

The patients’ baseline clinical characteristics are shown in Table 1. Patients with an hANP level ≥ 100 pg/mL tended to have lower DW, body mass index (BMI), and albumin levels than those with an hANP level < 100 pg/mL (Table 1). Additionally, age, post-dialysis systolic BP and the rate of past CVD tended to be higher in patients with an hANP level ≥ 100 pg/mL. A limited number of patients failed to achieve DW and the median difference between post-dialysis body weight and DW was 0.2 kg, which was not statistically significant between the two groups. There were no significant differences in medications, such as angiotensin-converting enzyme inhibitors, angiotensin receptor blockers, or β-blockers, between the two groups. During the dialysis sessions, 12 patients experienced hypoxemia due to congestion. After calculating the odds ratio, we confirmed that patients with an hANP level ≥ 100 pg/mL were at a significantly higher risk of developing hypoxemia due to congestion (Table 2). ROC curve analysis demonstrated that the threshold hANP level was 103 pg/mL in this short-term study. (Figure S1).

**Table 1:** The baseline clinical characteristics

	Overall (n = 156)	hANP ≥ 100 pg/mL (n = 48)	hANP < 100 pg/mL (n = 108)	*P value*
Male sex (n [%])	103 (66.0)	28 (58.3)	75 (69.4)	0.18
Age (years; mean ± SD, [range])	67 ± 13 (26–95)	72 ± 12 (45–95)	65 ± 13 (26–95)	0.002
DW (kg; mean ± SD)	55.2 ± 12.1	50.1 ± 9.5	57.4 ± 12.4	< 0.001
Difference between post-dialysis body weight and DW (kg; median [IQR])	0.2 (0–0.6)	0.3 (0–0.8)	0.2 (0–0.6)	0.32
Height (cm; median [IQR])	162 (153–169)	160 (150–167)	163 (155–169)	0.053
BMI (mean ± SD)	21.2 ± 3.3	19.9 ± 2.4	21.8 ± 3.5	0.001
Duration of dialysis (days; median [IQR])	2,453 (1,114–5,434)	2,173 (1,339–4,703)	2,532 (1,046–5,480)	0.81
Kt/V (median [IQR])	1.62 (1.46–1.81)	1.62 (1.46–1.79)	1.62 (1.45–1.83)	0.90
Ultrafiltration volume (L; mean ± SD)	3.0 ± 0.9	2.9 ± 0.9	3.0 ± 0.9	0.52
Dialysis session length (hours; mean ± SD)	4.1 ± 0.3	4.1 ± 0.3	4.1 ± 0.3	0.36
Ultrafiltration rate (L/h; mean ± SD)	0.7 ± 0.2	0.7 ± 0.2	0.7 ± 0.2	0.65
History of diabetes (n [%])	57 (36.5)	18 (37.5)	39 (36.1)	0.87
History of CVD (n [%])	72 (46.2)	30 (62.5)	42 (38.9)	0.006
History of respiratory diseases (n [%])	13 (8.3)	5 (10.4)	8 (7.4)	0.54
Pre-dialysis SBP (mmHg; mean ± SD)	152 ± 22	156 ± 23	150 ± 21	0.15
Post-dialysis SBP (mmHg; mean ± SD)	133 ± 22	143 ± 22	129 ± 21	< 0.001
Pre-dialysis DBP (mmHg; mean ± SD)	80 ± 15	80 ± 17	79 ± 15	0.84
Post-dialysis DBP (mmHg; mean ± SD)	73 ± 13	73 ± 13	73 ± 14	0.79
SpO_2_ (%; median [IQR])	98 (96–99)	97 (96–98)	98 (97–99)	0.001
Post-dialysis hANP (pg/mL; median [IQR])	57 (38–123)	166 (127–237)	46 (31–59)	< 0.001
Hemoglobin (g/dL; mean ± SD)	11.0 ± 1.0	10.6 ± 0.9	11.2 ± 0.9	0.001
CRP (mg/dL; median [IQR])	0.07 (0.03–0.25)	0.09 (0.03–0.35)	0.07 (0.03–0.19)	0.33
Total protein (g/dL; mean ± SD)	6.6 ± 0.5	6.5 ± 0.5	6.6 ± 0.5	0.18
Albumin (g/dL; median [IQR])	3.7 (3.5–3.9)	3.6 (3.5–3.8)	3.7 (3.5–3.9)	0.021
Pre-dialysis creatinine (mg/dL; mean ± SD)	10.7 ± 2.7	9.0 ± 2.2	11.4 ± 2.6	< 0.001
Modified creatinine index (mean ± SD)	20.5 ± 2.2	20.0 ± 2.3	20.8 ± 2.1	0.063
Use of ACE inhibitors or ARB (n [%])	84 (53.8)	21 (43.8)	63 (58.3)	0.092
Use of β-blocker (n [%])	33 (21.2)	13 (27.1)	20 (18.5)	0.23

*hANP* human atrial natriuretic peptide, *DW* dry weight, *BMI* body mass index, *CVD* cardiovascular disease, *SBP* systolic blood pressure, *DBP* diastolic blood pressure, *SpO*_*2*_ peripheral oxygen saturation, *CRP* C-reactive protein, *ACE* angiotensin-converting enzyme, *ARB* angiotensin receptor blocker, *SD* standard deviation, *IQR* interquartile range

**Table 2:** Odds ratio of hypoxemia due to congestion according to the hANP level

	hANP		OR (95% CI)	*P value*
	≥ 100 pg/mL (n = 48)	< 100 pg/mL (n = 108)		
Hypoxemia due to congestion (n [%])	7 (14.6)	5 (4.6)	3.52 (1.06–11.72)	0.040

*hANP* human atrial natriuretic peptide, *OR* odds ratio, *CI* confidence interval

### Clinical follow-up

**Fig. 1:**
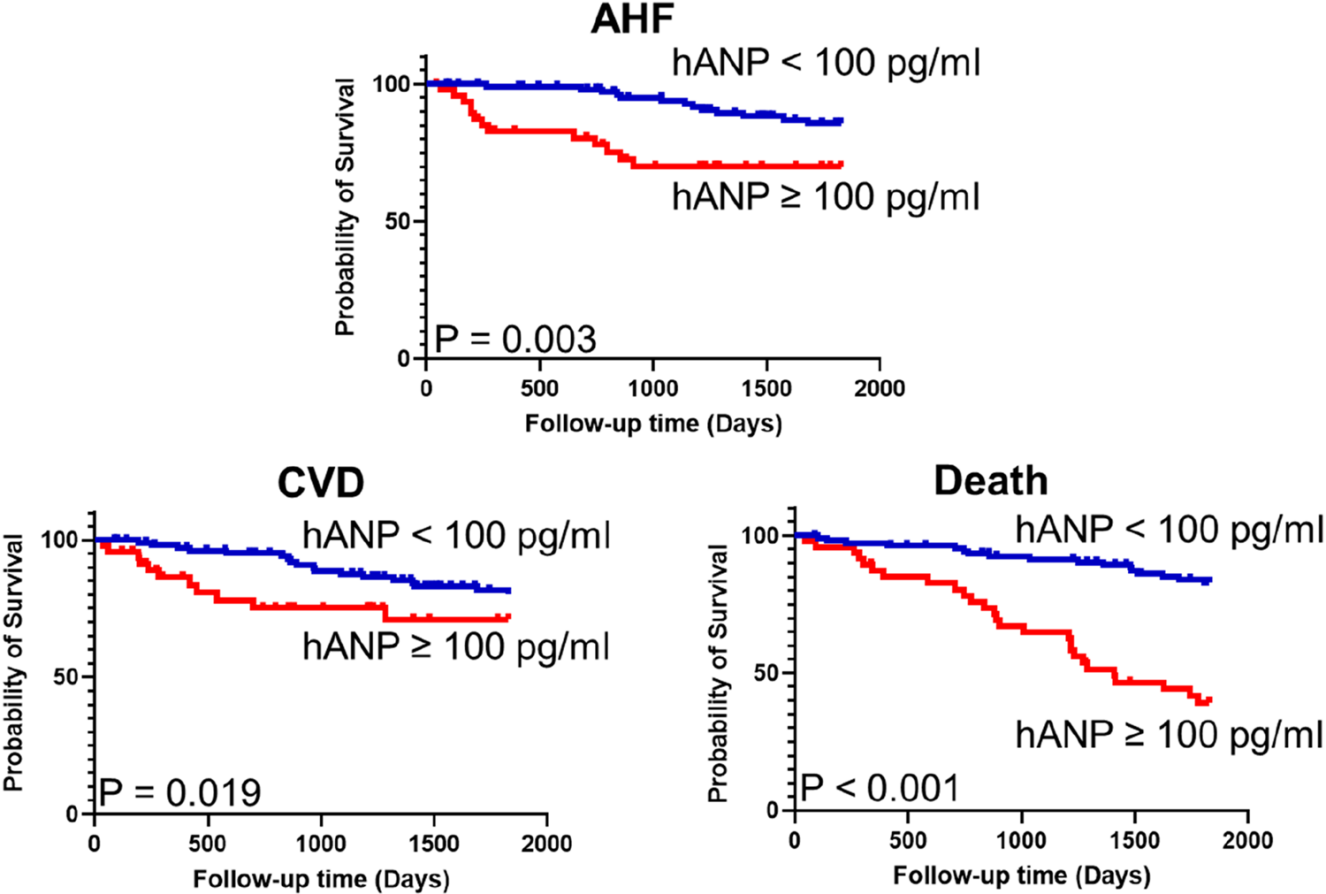
Kaplan–Meier analysis for hospitalization due to AHF, CVD development, and all-cause death at the 5-year clinical follow up. Patients were grouped according to their hANP level (≥ 100 pg/mL and < 100 pg/mL). The estimates were compared by using the log-rank test. *hANP* human atrial natriuretic peptide, *AHF* acute heart failure, *CVD* cardiovascular disease

**Fig. 2:**
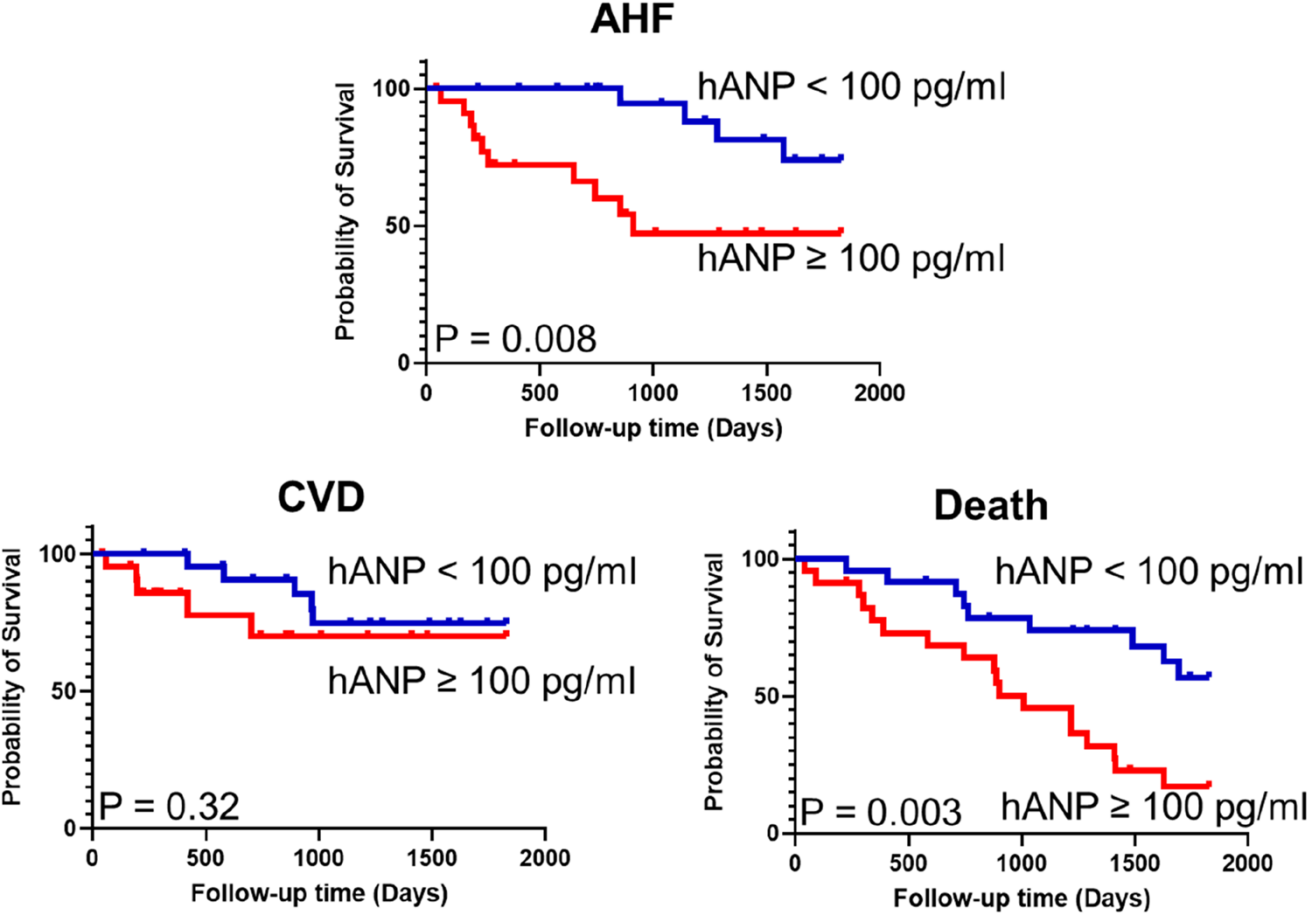
Kaplan–Meier analysis for hospitalization due to AHF, CVD development, and all-cause death at the 5-year clinical follow-up in patients aged ≥ 75 years. The patients were grouped according to their hANP level (≥ 100 pg/mL and < 100 pg/mL). The estimates were compared by using the log-rank test. *hANP* human atrial natriuretic peptide, *AHF* acute heart failure, *CVD* cardiovascular disease

During the 5-year follow-up, 26 patients were hospitalized due to AHF, 29 patients developed CVD, 44 patients died, and 20 patients were censored because they transferred hospitals. The Kaplan–Meier curves demonstrated that patients with an hANP level ≥ 100 pg/mL had a significantly higher rate of hospitalization due to AHF, CVD, and all-cause death than those with an hANP level < 100 pg/mL (Fig. 1). The sensitivity and specificity of the threshold hANP level for each outcome are presented in Table S1, and ROC curves are illustrated in Figure S1. The rates of death by HF, infection, and sudden death were significantly higher in patients with an hANP level ≥ 100 pg/mL (Table S2). Because patients with an hANP level ≥ 100 pg/mL were significantly older, we extracted data of elderly patients aged ≥ 75 years (Table 3). The baseline clinical characteristics and the cause of death in patients aged ≥ 75 years are shown in Table S3 and S4, respectively. Although the age of patients in both groups was not significantly different, elderly patients with an hANP level < 100 pg/mL had a lower rate of hospitalization due to AHF and all-cause death than those with an hANP level ≥ 100 pg/mL (Fig. 2). Cox proportional hazard model revealed that the hANP level remained independently associated with hospitalization due to AHF after adjusting for sex, age, hemoglobin, albumin, and history of diabetes (Table 4).

**Table 3:** The prognosis of the patients aged ≥ 75 years

	Overall (n = 47)	hANP ≥ 100 pg/mL (n = 23)	hANP < 100 pg/mL (n = 24)	*P value*
Age (years; mean ± SD)	82.1 ± 5.2	82.3 ± 5.3	82.0 ± 5.1	0.82
Hospitalized AHF (n [%])	14 (29.8)	10 (43.5)	4 (16.7)	0.042
CVD (n [%])	10 (21.3)	5 (21.7)	5 (20.8)	0.94
All-cause death (n [%])	27 (57.5)	18 (78.3)	9 (37.5)	0.004

*hANP* human atrial natriuretic peptide, *AHF* acute heart failure, *CVD* cardiovascular disease,* SD* standard deviation

**Table 4:** Impact of hANP and other predictive factors on hospitalization due to AHF by Cox regression models

Parameter	Hazard ratio	95% CI	*P value*
hANP of ≥ 100 pg/mL	3.00	1.33–6.79	0.010
Male sex	0.74	0.31–1.74	0.49
Age	1.05	1.01–1.10	0.008
Hemoglobin	1.81	1.07–3.14	0.026
Albumin	0.79	0.20–3.22	0.74
History of diabetes	2.81	1.24–6.36	0.013

hANP, human atrial natriuretic peptide; AHF, acute heart failure; CI, confidence interval

### Echocardiography

The echocardiography data of all patients is presented in Table 5 and that of the elderly patients is presented in Table S5. In patients with an hANP level ≥ 100 pg/mL, LVEF was significantly lower, left atrial diameter (LAD) and LVMI were significantly higher, compared to those with an hANP level < 100 pg/mL (Table 5). Additionally, the proportions of patients with diastolic dysfunction and those with HFpEF were also significantly higher in patients with an hANP level ≥ 100 pg/mL (Table 5). The baseline clinical characteristics according to cardiac functions are detailed in Table S6. More than 70% of patients with HFpEF or systolic dysfunction presented with an hANP level ≥ 100 pg/mL (Table S6). When comparing prognosis, systolic dysfunction and HFpEF were significantly associated with all-cause death at the 5-year clinical follow-up (Figure S2). The rate of hospitalization due to AHF tended to be higher in patients with systolic dysfunction and those with HFpEF, however, there was no significant difference (Figure S2). When conducting Cox proportional hazard model analysis with hANP level and cardiac function, only an hANP level ≥ 100 pg/mL was significantly associated with hospitalization due to AHF (Table 6). Figure S3 illustrates the impacts of hANP on prognosis in each group divided by cardiac functions. In patients with normal cardiac function, an hANP level ≥ 100 pg/mL was significantly related to a higher rate of hospitalization due to AHF and all-cause death. In patients with HFpEF or systolic dysfunction, the rate of developing AHF tended to be higher in patients with an hANP level ≥ 100 pg/mL, however, the differences were not significant.

**Table 5:** Echocardiographic data

	Overall (n = 154)	hANP ≥ 100 pg/mL (n = 47)	hANP < 100 pg/mL (n = 107)	*P value*
LVEF (%; median [IQR])	62 (56–66)	57 (43–63)	63 (58–67)	< 0.001
Systolic disfunction (n [%])	19 (12.3)	15 (31.3)	4 (3.8)	< 0.001
Normal diastolic function (n [%])	104 (67.5)	18 (37.5)	86 (81.1)	< 0.001
Indeterminate diastolic function (n [%])	22 (14.3)	8 (16.7)	14 (13.2)	0.57
Diastolic disfunction (n [%])	28 (18.2)	22 (45.8)	6 (5.7)	< 0.001
HFpEF (n [%])	9 (5.8)	7 (14.6)	2 (1.9)	0.003
LAD (mm; mean ± SD)	40.7 ± 5.9	43.1 ± 6.3	39.6 ± 5.4	< 0.001
LVMI (g/m^2^; median [IQR])	129 (108–159)	158 (130–194)	116 (104–144)	< 0.001
IVC diameter (mm; mean ± SD)	15.2 ± 3.4	15.9 ± 3.9	14.8 ± 3.1	0.075
IVST (mm; median [IQR])	11 (10–12)	11 (10–13)	11 (10–12)	0.039
PWT (mm; median [IQR])	11 (10–12)	11 (10–12)	10 (10–12)	0.008
LVDd (mm; median [IQR])	49 (45–53)	51 (47–57)	48 (44–52)	0.005
LVDs (mm; median [IQR])	32 (29–37)	37 (32–43)	32 (29–35)	< 0.001
Peak E-wave velocity (cm/sec; median [IQR])	81 (65–102)	99 (67–111)	75 (64–93)	0.003
Lateral e’ velocity (cm/sec; median [IQR])	7.9 (6.4–10.1)	7.1 (5.7–8.8)	8.5 (6.8–11.0)	< 0.001
Mitral E/A ratio (median [IQR])	0.84 (0.69–1.01)	0.86 (0.68–1.31)	0.83 (0.69–0.94)	0.16
Mitral E/e’ ratio (median [IQR])	9.6 (7.1–13.3)	13.0 (9.3–18.1)	9.4 (6.9–10.6)	< 0.001
TRPG (mmHg; median [IQR])	27 (21–33)	29 (23–40)	25 (20–30)	0.004

*hANP* human atrial natriuretic peptide, *LVEF* left ventricular ejection fraction, *HFpEF* heart failure with preserved ejection fraction, *LAD* left atrial diameter, *LVMI* left ventricular mass index, *IVC* inferior vena cava, *IVST* interventricular septum thickness, *PWT* posterior left ventricular wall thickness, *LVDd* left ventricular end-diastolic dimension, *LVDs* left ventricular end-systolic dimension, *E/A rate* the ratio of the early to late ventricular filling velocities, *E/e’* *rate* the ratio of mitral peak velocity of early filling to early diastolic mitral annular velocity, *TRPG* tricuspid regurgitation peak gradient, *SD* standard deviation, *IQR* interquartile range

**Table 6:** Impact of hANP and cardiac functions on hospitalization due to AHF by Cox regression models

Parameter	Hazard ratio	95% CI	*P value*
hANP of ≥ 100 pg/mL	2.56	1.07–6.12	0.034
HFpEF vs. Normal cardiac function	1.61	0.34–7.57	0.55
Systolic disfunction vs. Normal cardiac function	1.44	0.49–4.23	0.51
Systolic disfunction vs. HFpEF	0.90	0.17–4.63	0.90

*hANP* human atrial natriuretic peptide, *AHF* acute heart failure, *HFpEF* heart failure with preserved ejection fraction, *CI* confidence interval

## Discussion

This study showed that the high plasma hANP level reflects volume overload, which leads to hypoxemia due to congestion. In addition, we revealed that a high plasma hANP level was significantly related to hospitalization due to AHF, CVD, and all-cause death in patients on hemodialysis. Analysis of echocardiography data revealed that cardiac dysfunctions were significantly associated with hANP. However, the threshold hANP level (100 pg/mL) remained independently associated with hospitalization due to AHF after adjusting for cardiac disfunctions in the multivariable model.

In the short-term observation, our results showed that high level of hANP reflected volume overload and was associated with hypoxemia due to congestion. Although many studies have shown a relationship between hANP and fluid volume [6, 17, 18], few studies have reported on the threshold hANP level. The Japanese Society for Dialysis Therapy Guidelines reported a threshold hANP level of 50–100 pg/mL when DW is achieved [11]. The threshold level is estimated by other markers for fluid volume such as edema, blood pressure, CTR, and left atrial dimension. However, no studies had evaluated the usefulness of the threshold in the short-term clinical outcome. Our results support the usefulness of 100 pg/mL as the threshold for assessing the risk of developing congestion.

Our 5-year follow-up study revealed that patients with an hANP level ≥ 100 pg/mL had a significantly higher rate of hospitalization due to AHF, CVD, and all-cause death. In addition, the Cox proportional hazard model showed that hANP was an independent prognostic marker of hospitalization due to AHF after adjusting for sex, age, hemoglobin, albumin, and history of diabetes. We consider that the patients with an hANP level ≥ 100 pg/mL have either or both of temporary volume overload and cardiac disfunctions, and they are vulnerable to the development of AHF, which worsens the prognosis. The mortality in patients with AHF is reportedly high [19], and admission for AHF leads to worse prognosis [20]. Patients with an hANP level ≥ 100 pg/mL had significantly higher rates of death caused by HF and sudden death, which are the major causes of death for patients who have been admitted due to AHF [19]. Although the relationship between hANP and mortality has been reported [7–10], this is the first study that showed that a high hANP level may predict hospitalization due to AHF. Based on this result, the threshold hANP level of 100 pg/mL is valuable not only for short-term fluid volume assessment but also for predicting long-term prognosis.

Patients with an hANP level ≥ 100 pg/mL were older and had a lower DW and BMI, which may be attributed to muscle attenuation and change in body fluid distribution as a result of aging. Although both intracellular (ICW) and extracellular water (ECW) volume decrease with aging, the ECW/ICW ratio reportedly increases in elderly patients on hemodialysis [21]. Approximately 75% of muscle volume is composed of water [22]; thus, lower DW and BMI in elderly patients with an hANP level ≥ 100 pg/mL may reflect decreased muscle mass and ICW. High plasma hANP level in elderly patients may possibly be caused by the relatively higher ratio of ECW, which leads to the susceptibility to volume overload. In addition, the patients with cardiac disfunctions were older than those with normal cardiac function, which could be another reason of high plasma hANP level in elderly patients. As the plasma hANP level predicted the development of AHF and all-cause death in patients aged > 75 years, it may be used as a prognostic marker in elderly people.

Analysis of the echocardiography data revealed that systolic dysfunction was significantly associated with the hANP level, consistent with the findings of previous studies [8, 9]. This study also demonstrated that left ventricular diastolic dysfunction was also associated with the hANP level, which has not been previously reported. Left atrial dysfunction and dilated left atrium were reportedly associated with HFpEF [14, 15, 23], thus it is plausible that increased atrial wall stress leads to a high plasma hANP level. In patients with HFpEF or systolic dysfunction, over 70% of them exhibited hANP levels ≥ 100 pg/mL, and there were no significant differences in prognosis between two groups divided by the threshold hANP level. This result indicates that it is uncertain whether lowering the hANP level below the threshold can improve the prognosis of patients with cardiac dysfunction. On the other hand, the threshold hANP level was more strongly associated with hospitalization due to AHF than the cardiac dysfunctions. A possible explanation for this is that the hANP level reflects not only cardiac functions but also volume overload. As illustrated in Figure S3, the threshold hANP level predicted the development of AHF in patients with normal cardiac function. Based on these results, we consider that hANP can serve as a simple and convenient marker to identify high-risk patients for developing AHF, as well as a fluid volume marker for patients with normal cardiac function.

Several limitations need to be considered when interpreting our results. First, this study is a single-center observational study; thus, there could be selection bias. Second, we were unable to demonstrate the superiority of hANP compared to other measures of fluid volume assessment, such as BNP, as we did not have data of them at the same point of hANP measurement. Third, although we measured the plasma hANP level at the start of the study, we did not routinely measure the hANP level of all the patients during the clinical follow-up. For this reason, we could not follow the changes of the hANP level, and whether controlling hANP can improve prognosis or not cannot be deduced from this study. To evaluate the effectiveness of controlling hANP in improving prognosis, further multicenter prospective studies should be conducted.

In conclusion, our study demonstrated the utility of the threshold hANP level in both short-term and long-term clinical outcomes. In clinical practice, we suggest utilizing hANP as an easily accessible measure to identify high-risk patients for developing AHF initially. If the hANP level exceeds 100 mg/dL, we propose to perform echocardiography and consider reducing the DW. In clinical practice, the plasma hANP level could serve as a valuable indicator for assessing the risk of developing AHF and managing fluid volume.

## Supplementary Information

Below is the link to the electronic supplementary material.

Supplementary Fig. S1 ROC curve analysis of hANP in predicting hypoxia due to congestion at the short term study, hospitalization for acute heart failure, development of cardiovascular disease, and all-cause mortality at 5-year clinical follow-up. ROC, receiver operating characteristics; hANP, human atrial natriuretic peptide; AHF, acute heart failure; CVD, cardiovascular diseas; AUC, area under the curve file1 (DOCX 138 KB)

Supplementary Fig. S2 Kaplan–Meier analysis for hospitalization due to AHF, CVD development, and all-cause death at the 5-year clinical follow up. Patients were grouped according to their cardiac functions (LVEF ≥ 50% vs. LVEF < 50%, normal cardiac function vs. HFpEF, or normal cardiac function vs. HFpEF vs. systolic disfunction). The estimates were compared by using the log-rank test. LVEF, left ventricular ejection fraction; HFpEF, heart failure with preserved ejection fraction; AHF, acute heart failure; CVD, cardiovascular disease file2 (DOCX 93 KB)

Supplementary Kaplan–Meier analysis for hospitalization due to AHF, CVD development, and all-cause death at the 5-year clinical follow-up according to the hANP level and cardiac functions. The patients were grouped according to their hANP level (≥ 100 pg/mL and < 100 pg/mL) in each group divided by cardiac functions (normal cardiac function, HFpEF, systolic dsifunction). The estimates were compared by using the log-rank test. hANP, human atrial natriuretic peptide; AHF, acute heart failure; CVD, cardiovascular disease, HFpEF, heart failure with preserved ejection fraction file3 (DOCX 120 KB)

Supplementary file4 (DOCX 39 KB)

## Data Availability

The data that support the findings of this study are available from the corresponding author, TO, upon reasonable request.
