# Supplementary material for: Assessing fluid volume and determining outcomes of acute heart failure using plasma human atrial natriuretic peptide
Source: Clin Exp Nephrol. 2023 Mar 20;27(6):565–73. doi: 10.1007/s10157-023-02333-1 (PMC10191894; doi:10.1007/s10157-023-02333-1)

**Supplementary Figure S1**

**Title:**

Assessing fluid volume and determining outcomes of acute heart failure using plasma human atrial natriuretic peptide

Yuya Suzuki^1^, Tadashi Otsuka^1^, Yuki Yoshioka^2^, Tomomichi Iida^3^, Shingo Maruyama^1^, Hirofumi Watanabe^1^, Ryohei Kaseda^1^, Suguru Yamamoto^1^, Yoshikatsu Kaneko^1^, Shin Goto^1^, Ryuji Aoyagi^3^, Ichiei Narita^1^

^1^Division of Clinical Nephrology and Rheumatology, Kidney Research Center, Niigata University Graduate School of Medical and Dental Sciences, Niigata, Japan

^2^Division of Nephrology and Hypertension, Department of Internal Medicine, The Jikei University Daisan Hospital, Tokyo, Japan

^3^Department of Nephrology, Tachikawa General Hospital, Niigata, Japan

**Journal name:**

Clinical and Experimental Nephrology

**Corresponding Author:**

Tadashi Otsuka, M.D., Ph.D.

1-757 Asahimachi, Chuo-ku, Niigata 951-8510, Japan

E-mail: totogoal1230@gmail.com

**Fig. S1**


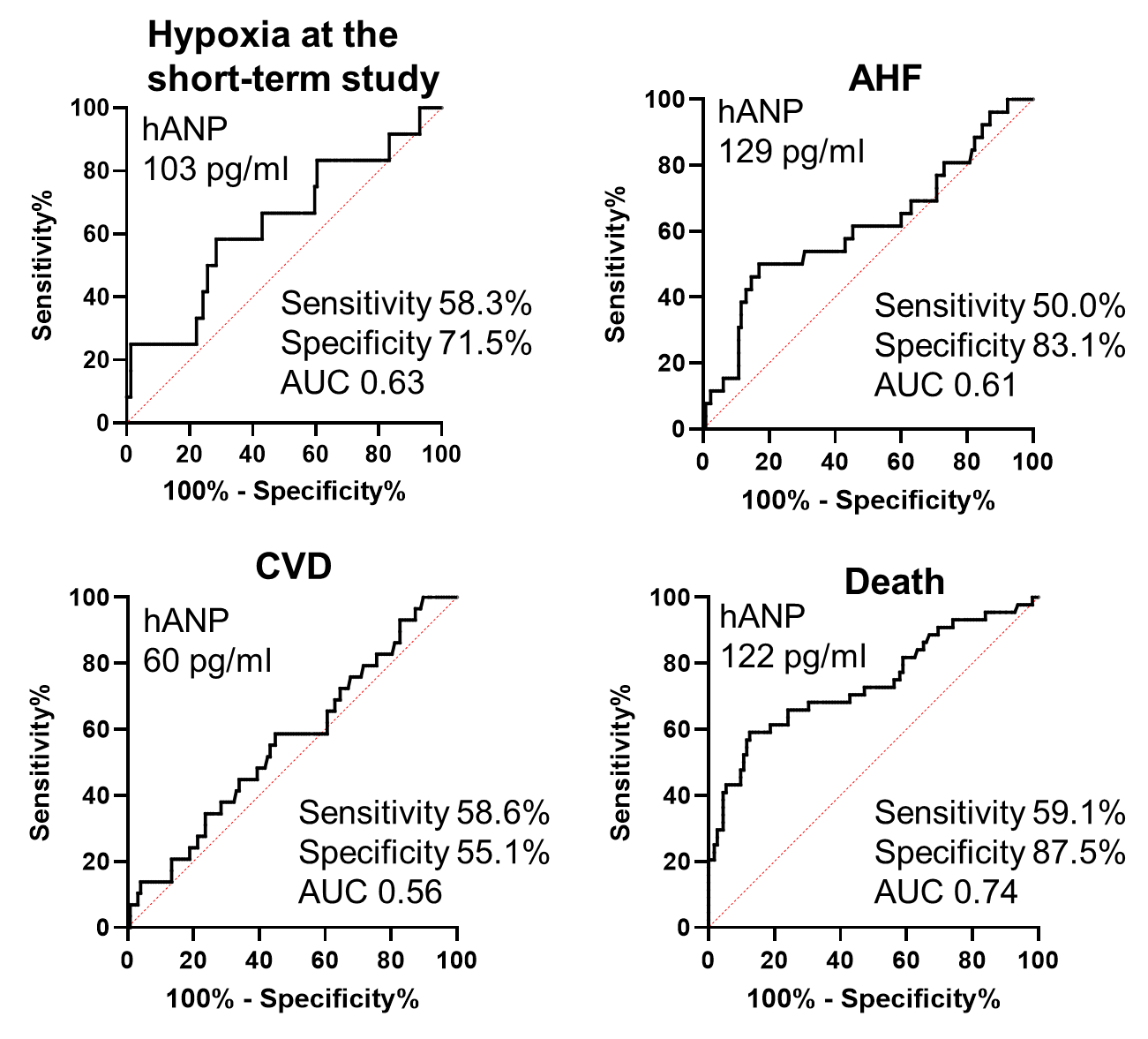

Supplement: Supplementary file 1 — Supplementary Fig. S1 ROC curve analysis of hANP in predicting hypoxia due to congestion at the short term study, hospitalization for acute heart failure, development of cardiovascular disease, and all-cause mortality at 5-year clinical follow-up. ROC, receiver operating characteristics; hANP, human atrial natriuretic peptide; AHF, acute heart failure; CVD, cardiovascular diseas; AUC, area under the curve file1 (DOCX 138 KB) [file 10157_2023_2333_MOESM1_ESM.docx]
