# Supplementary material for: Assessing fluid volume and determining outcomes of acute heart failure using plasma human atrial natriuretic peptide
Source: Clin Exp Nephrol. 2023 Mar 20;27(6):565–73. doi: 10.1007/s10157-023-02333-1 (PMC10191894; doi:10.1007/s10157-023-02333-1)

**Supplementary Figure S2**

**Title:**

Assessing fluid volume and determining outcomes of acute heart failure using plasma human atrial natriuretic peptide

Yuya Suzuki^1^, Tadashi Otsuka^1^, Yuki Yoshioka^2^, Tomomichi Iida^3^, Shingo Maruyama^1^, Hirofumi Watanabe^1^, Ryohei Kaseda^1^, Suguru Yamamoto^1^, Yoshikatsu Kaneko^1^, Shin Goto^1^, Ryuji Aoyagi^3^, Ichiei Narita^1^

^1^Division of Clinical Nephrology and Rheumatology, Kidney Research Center, Niigata University Graduate School of Medical and Dental Sciences, Niigata, Japan

^2^Division of Nephrology and Hypertension, Department of Internal Medicine, The Jikei University Daisan Hospital, Tokyo, Japan

^3^Department of Nephrology, Tachikawa General Hospital, Niigata, Japan

**Journal name:**

Clinical and Experimental Nephrology

**Corresponding Author:**

Tadashi Otsuka, M.D., Ph.D.

1-757 Asahimachi, Chuo-ku, Niigata 951-8510, Japan

E-mail: totogoal1230@gmail.com

**Fig. S2**


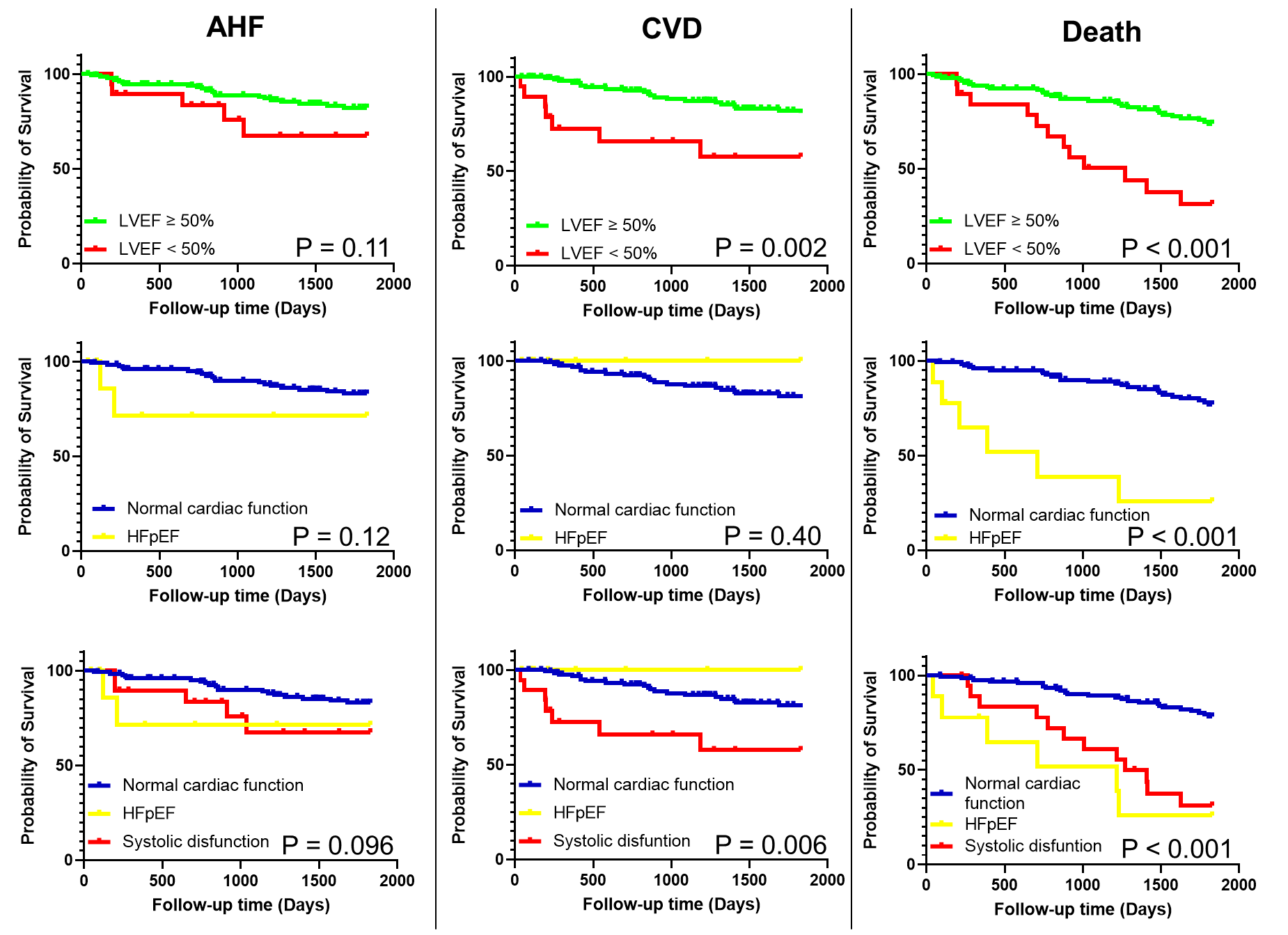

Supplement: Supplementary file 2 — Supplementary Fig. S2 Kaplan–Meier analysis for hospitalization due to AHF, CVD development, and all-cause death at the 5-year clinical follow up. Patients were grouped according to their cardiac functions (LVEF ≥ 50% vs. LVEF < 50%, normal cardiac function vs. HFpEF, or normal cardiac function vs. HFpEF vs. systolic disfunction). The estimates were compared by using the log-rank test. LVEF, left ventricular ejection fraction; HFpEF, heart failure with preserved ejection fraction; AHF, acute heart failure; CVD, cardiovascular disease file2 (DOCX 93 KB) [file 10157_2023_2333_MOESM2_ESM.docx]
