# Supplementary material for: Assessing fluid volume and determining outcomes of acute heart failure using plasma human atrial natriuretic peptide
Source: Clin Exp Nephrol. 2023 Mar 20;27(6):565–73. doi: 10.1007/s10157-023-02333-1 (PMC10191894; doi:10.1007/s10157-023-02333-1)
Supplement: Supplementary file 4 — Supplementary file4 (DOCX 39 KB) [file 10157_2023_2333_MOESM4_ESM.docx]

**Supplementary Tables**

**Title:**

Assessing fluid volume and determining outcomes of acute heart failure using plasma human atrial natriuretic peptide

Yuya Suzuki^1^, Tadashi Otsuka^1^, Yuki Yoshioka^2^, Tomomichi Iida^3^, Shingo Maruyama^1^, Hirofumi Watanabe^1^, Ryohei Kaseda^1^, Suguru Yamamoto^1^, Yoshikatsu Kaneko^1^, Shin Goto^1^, Ryuji Aoyagi^3^, Ichiei Narita^1^

^1^Division of Clinical Nephrology and Rheumatology, Kidney Research Center, Niigata University Graduate School of Medical and Dental Sciences, Niigata, Japan

^2^Division of Nephrology and Hypertension, Department of Internal Medicine, The Jikei University Daisan Hospital, Tokyo, Japan

^3^Department of Nephrology, Tachikawa General Hospital, Niigata, Japan

**Journal name:**

Clinical and Experimental Nephrology

**Corresponding Author:**

Tadashi Otsuka, M.D., Ph.D.

1-757 Asahimachi, Chuo-ku, Niigata 951-8510, Japan

E-mail: totogoal1230@gmail.com

|  |  | Sensitivity | |  | Specificity | |
| --- | --- | --- | --- | --- | --- | --- |
| Cut-off value of hANP |  | 50 pg/mL | 100 pg/mL |  | 50 pg/mL | 100 pg/mL |
| Hospitalized AHF (%) |  | 65.4 | 50.0 |  | 40.0 | 73.1 |
| CVD (%) |  | 62.1 | 37.9 |  | 39.4 | 70.9 |
| All-cause death (%) |  | 72.7 | 61.4 |  | 43.8 | 81.3 |

**Table S1**. The sensitivity and specificity of the threshold hANP level (50 pg/mL or 100 pg/mL) in relation to hospitalized AHF, CVD, and all-cause death

hANP, human atrial natriuretic peptide; AHF, acute heart failure; CVD, cardiovascular disease

|  | Overall (n = 156) | hANP ≥ 100 pg/mL (n = 48) | hANP < 100 pg/mL (n = 108) | P value |
| --- | --- | --- | --- | --- |
| HF (n [%]) | 7 (4.5) | 6 (12.5) | 1 (0.9) | 0.004 |
| AMI (n [%]) | 3 (1.9) | 2 (4.2) | 1 (0.9) | 0.22 |
| Cerebral infarction or Cerebral hemorrhage　(n [%]) | 3 (1.9) | 2 (4.2) | 1 (0.9) | 0.22 |
| NOMI (n [%]) | 4 (2.6) | 3 (6.3) | 1 (0.9) | 0.087 |
| Infection (n [%]) | 7 (4.5) | 5 (10.4) | 2 (1.9) | 0.029 |
| Malignancy (n [%]) | 6 (3.8) | 1 (2.1) | 5 (4.6) | 0.67 |
| Sudden death (n [%]) | 6 (3.8) | 5 (10.4) | 1 (0.9) | 0.011 |
| Others (n [%]) | 8 (5.1) | 5 (10.4) | 3 (2.8) | 0.059 |

**Table S2**. The cause of death during the 5-year follow-up

hANP, human atrial natriuretic peptide; HF, heart failure; AMI, acute myocardial infarction; NOMI, non-occlusive mesenteric ischemia

**Table S3**. The baseline clinical characteristics of the patients aged ≥ 75 years

|  | Overall (n = 47) | hANP ≥ 100 pg/mL (n = 23) | hANP < 100 pg/mL (n = 24) | P value |
| --- | --- | --- | --- | --- |
| Male sex (n [%]) | 22 (46.8) | 11 (47.8) | 11 (45.8) | 0.89 |
| DW (kg; mean ± SD) | 48.6 ± 8.9 | 47.8 ± 8.2 | 49.5 ± 9.6 | 0.52 |
| Difference between post-dialysis body weight and DW (kg; median [IQR]) | 0.3 (0-0.5) | 0.3 (0-0.4) | 0.3 (0-0.6) | 0.64 |
| Height (cm; median [IQR]) | 157 (146–162) | 156 (145–164) | 159 (147–162) | 0.88 |
| BMI (mean ± SD) | 20.3 ± 2.6 | 20.1 ± 2.3 | 20.6 ± 2.9 | 0.53 |
| Duration of dialysis (days; median [IQR]) | 2,107 (802–2,992) | 1,831 (802–2,992) | 2,322 (764–3,458) | 0.91 |
| Kt/V (median [IQR]) | 1.63 (1.48–1.86) | 1.61 (1.47–1.79) | 1.65 (1.56–1.90) | 0.34 |
| Ultrafiltration volume (L; mean ± SD) | 2.5 ± 0.6 | 2.4 ± 0.5 | 2.5 ± 0.7 | 0.83 |
| Dialysis session length (hours; mean ± SD) | 4.0 ± 0.2 | 4.0 ± 0.2 | 4.0 ± 0.1 | 0.67 |
| Ultrafiltration rate (L/h; mean ± SD) | 0.6 ± 0.2 | 0.6 ± 0.1 | 0.6 ± 0.2 | 0.87 |
| History of diabetes (n [%]) | 18 (38.3) | 8 (34.8) | 10 (41.7) | 0.63 |
| History of CVD (n [%]) | 33 (70.2) | 18 (78.3) | 15 (62.5) | 0.23 |
| History of respiratory diseases (n [%]) | 6 (12.8) | 4 (17.4) | 2 (8.3) | 0.35 |
| Pre-dialysis SBP (mmHg; mean ± SD) | 156 ± 21 | 161 ± 21 | 151 ± 20 | 0.093 |
| Post-dialysis SBP (mmHg; mean ± SD) | 136 ± 22 | 143 ± 19 | 130 ± 24 | 0.058 |
| Pre-dialysis DBP (mmHg; mean ± SD) | 73 ± 14 | 77 ± 16 | 69 ± 11 | 0.051 |
| Post-dialysis DBP (mmHg; mean ± SD) | 68 ± 11 | 70 ± 10 | 66 ± 12 | 0.22 |
| SpO_2_ (%; median [IQR]) | 97 (96–98) | 97 (96–98) | 97 (97–98) | 0.36 |
| Post-dialysis hANP  (pg/mL; median [IQR]) | 93 (47–185) | 185 (137–233) | 48 (39–73) | < 0.001 |
| Hemoglobin (g/dL; mean ± SD) | 10.6 ± 0.8 | 10.5 ± 0.7 | 10.7 ± 0.8 | 0.33 |
| CRP (mg/dL; median [IQR]) | 0.09 (0.03–0.30) | 0.18 (0.04–0.39) | 0.06 (0.02–0.18) | 0.23 |
| Total protein (g/dL; mean ± SD) | 6.6 ± 0.5 | 6.4 ± 0.5 | 6.7 ± 0.5 | 0.052 |
| Albumin (g/dL; median [IQR]) | 3.6 (3.4–3.7) | 3.5 (3.4–3.6) | 3.7 (3.6–4.0) | 0.008 |
| Pre-dialysis creatinine  (mg/dL; mean ± SD) | 8.9 ± 2.0 | 8.1 ± 1.4 | 9.7 ± 2.1 | 0.004 |
| Modified creatinine index (mean ± SD) | 20.2 ± 2.2 | 19.6 ± 2.2 | 20.7 ± 2.1 | 0.075 |
| Use of ACE inhibitors or ARB (n [%]) | 23 (48.9) | 6 (26.1) | 17 (70.8) | 0.002 |
| Use of β-blocker (n [%]) | 11 (23.4) | 7 (30.4) | 4 (16.7) | 0.26 |

hANP, human atrial natriuretic peptide; DW, dry weight; BMI, body mass index; CVD, cardiovascular disease; SBP, systolic blood pressure; DBP, diastolic blood pressure; SpO_2,_ peripheral oxygen saturation, CRP, C-reactive protein; ACE, angiotensin-converting enzyme; ARB, angiotensin receptor blocker; SD, standard deviation; IQR, interquartile range

**Table S4**. The cause of death in the patients aged ≥ 75 years during the 5-year follow-up

|  | Overall (n = 47) | hANP ≥ 100 pg/mL (n = 23) | hANP < 100 pg/mL (n = 24) | P value |
| --- | --- | --- | --- | --- |
| HF (n [%]) | 4 (8.5) | 3 (13.0) | 1 (4.2) | 0.35 |
| AMI (n [%]) | 2 (4.3) | 1 (4.3) | 1 (4.2) | 1.0 |
| Cerebral infarction or Cerebral hemorrhage (n [%]) | 2 (4.3) | 1 (4.3) | 1 (4.2) | 1.0 |
| NOMI (n [%]) | 3 (6.4) | 3 (13.0) | 0 (0) | 0.11 |
| Infection (n [%]) | 7 (14.9) | 5 (21.7) | 2 (8.3) | 0.24 |
| Malignancy (n [%]) | 3 (6.4) | 1 (4.3) | 2 (8.3) | 1.0 |
| Sudden death (n [%]) | 1 (2.1) | 1 (4.3) | 0 (0) | 0.49 |
| Others (n [%]) | 5 (10.6) | 2 (8.7) | 3 (12.5) | 1.0 |

hANP, human atrial natriuretic peptide; HF, heart failure; AMI, acute myocardial infarction; NOMI, non-occlusive mesenteric ischemia

**Table S5**. Echocardiographic data of the patients aged ≥ 75 years

|  | Overall (n = 46) | hANP ≥ 100 pg/mL (n = 23) | hANP < 100 pg/mL (n = 23) | P value |
| --- | --- | --- | --- | --- |
| LVEF (%; median [IQR]) | 61 (52–67) | 53 (42–62) | 64 (59–68) | < 0.001 |
| Systolic disfunction (n [%]) | 9 (19.6) | 9 (39.1) | 0 (0) | < 0.001 |
| Normal diastolic function | 20 (43.5) | 6 (26.1) | 14 (60.9) | 0.016 |
| Indeterminate diastolic function (n [%]) | 12 (26.1) | 4 (17.4) | 8 (34.8) | 0.18 |
| Diastolic disfunction (n [%]) | 14 (30.4) | 13 (56.5) | 1 (4.4) | < 0.001 |
| HFpEF (n [%]) | 5 (10.9) | 4 (17.4) | 1 (4.4) | 0.14 |
| LAD (mm; mean ± SD) | 40.9 ± 4.6 | 41.8 ± 4.9 | 39.9 ± 4.2 | 0.18 |
| LVMI (g/m^2^; median [IQR]) | 136 (110–162) | 152 (131–180) | 111 (107–143) | < 0.001 |
| IVC diameter (mm; mean ± SD) | 14.5 ± 2.7 | 14.8 ± 2.7 | 14.1 ± 2.7 | 0.40 |
| IVST (mm; median [IQR]) | 11 (10–12) | 11 (10–12) | 11 (10–12) | 0.11 |
| PWT (mm; median [IQR]) | 11 (10–12) | 11 (10–12) | 10 (9–11) | 0.021 |
| LVDd (mm; median [IQR]) | 48 (44–52) | 51 (45–56) | 47 (44–49) | 0.045 |
| LVDs (mm; median [IQR]) | 31 (28–39) | 38 (28–46) | 30 (27–32) | 0.008 |
| Peak E-wave velocity (cm/sec; median [IQR]) | 92 (68-111) | 101 (69-118) | 76 (64-103) | 0.14 |
| Lateral e’ velocity (cm/sec; median [IQR]) | 6.6 (5.8-8.1) | 6.2 (5.1-8.2) | 7.1 (6.4-8.3) | 0.068 |
| Mitral E/A ratio (median [IQR]) | 0.78 (0.66-0.92) | 0.78 (0.67-1.31) | 0.78 (0.64-0.89) | 0.29 |
| Mitral E/e’ ratio (median [IQR]) | 11.1 (9.5-18.6) | 15.3 (10.7-22.0) | 9.8 (8.7-13.2) | 0.009 |
| TRPG (mmHg; median [IQR]) | 33 (27-40) | 33 (25-45) | 32 (28-34) | 0.48 |

hANP, human atrial natriuretic peptide; LVEF, left ventricular ejection fraction; HFpEF, heart failure with preserved ejection fraction; LAD, left atrial diameter; LVMI, left ventricular mass index; IVC, inferior vena cava; IVST, interventricular septum thickness; PWT, posterior left ventricular wall thickness; LVDd, left ventricular end-diastolic dimension; LVDs, left ventricular end-systolic dimension; E/A rate, the ratio of the early to late ventricular filling velocities; E/e’ rate, the ratio of mitral peak velocity of early filling to early diastolic mitral annular velocity; TRPG, tricuspid regurgitation peak gradient; SD, standard deviation; IQR, interquartile range

**Table S6**. The baseline clinical characteristics of the patients grouped according to their cardiac functions

|  | Normal cardiac function (n = 126) | HFpEF (n = 9) | Systolic disfunction (n = 19) | P value |
| --- | --- | --- | --- | --- |
| Post-dialysis hANP (pg/mL; median [IQR]) | 54 (36–93) | 167 (109–214) | 170 (127–266) | < 0.001 |
| hANP ≥ 100 pg/mL (n [%]) | 26 (20.6) | 7 (77.8) | 15 (79.0) | < 0.001 |
| Male sex (n [%]) | 86 (68.3) | 5 (55.6) | 11 (57.9) | 0.54 |
| Age (years; mean ± SD, [range]) | 66 ± 13 (26–95) | 77 ± 13 (60–95) | 71 ± 11 (45–91) | 0.013 |
| DW (kg; mean ± SD) | 55.9 ± 12.4 | 49.2 ± 9.4 | 53.8 ± 10.3 | 0.25 |
| Difference between post-dialysis body weight and DW (kg; median [IQR]) | 0.2 (0-0.6) | 0.6 (0-1.3) | 0.1 (-0.1-0.8) | 0.37 |
| Height (cm; median [IQR]) | 163 (153–169) | 164 (149–170) | 157 (150–163) | 0.15 |
| BMI (mean ± SD) | 21.3 ± 3.3 | 19.4 ± 2.8 | 21.7 ± 2.8 | 0.19 |
| Duration of dialysis (days; median [IQR]) | 2,592 (1,046–5,381) | 1,904 (880–5,327) | 2,107 (1,333–4,420) | 0.95 |
| Kt/V (median [IQR]) | 1.62 (1.44–1.84) | 1.53 (1.42–1.71) | 1.63 (1.53–1.70) | 0.54 |
| Ultrafiltration volume (L; mean ± SD) | 3.0 ± 0.9 | 2.8 ± 0.9 | 3.0 ± 1.0 | 0.83 |
| Dialysis session length (hours; mean ± SD) | 4.1 ± 0.3 | 3.9 ± 0.2 | 4.2 ± 0.3 | 0.011 |
| Ultrafiltration rate (L/h; mean ± SD) | 0.7 ± 0.2 | 0.7 ± 0.2 | 0.7 ± 0.2 | 0.99 |
| History of diabetes (n [%]) | 49 (38.9) | 3 (33.3) | 5 (26.3) | 0.54 |
| History of CVD (n [%]) | 53 (42.1) | 4 (44.4) | 14 (73.7) | 0.033 |
| History of respiratory diseases (n [%]) | 11 (8.6) | 0 (0) | 2 (10.5) | 0.86 |
| Pre-dialysis SBP (mmHg; mean ± SD) | 151 ± 21 | 161 ± 35 | 157 ± 22 | 0.24 |
| Post-dialysis SBP (mmHg; mean ± SD) | 133 ± 22 | 146 ± 31 | 130 ± 16 | 0.19 |
| Pre-dialysis DBP (mmHg; mean ± SD) | 80 ± 16 | 78 ± 16 | 81 ± 14 | 0.84 |
| Post-dialysis DBP (mmHg; mean ± SD) | 74 ± 13 | 66 ± 11 | 72 ± 13 | 0.18 |
| SpO_2_ (%; median [IQR]) | 98 (97–99) | 97 (96–98) | 97 (96–98) | 0.028 |
| Hemoglobin (g/dL; mean ± SD) | 11.1 ± 0.9 | 10.1 ± 1.1 | 10.9 ± 1.0 | 0.006 |
| CRP (mg/dL; median [IQR]) | 0.06 (0.03–0.20) | 0.42 (0.05–0.73) | 0.13 (0.05–0.30) | 0.053 |
| Total protein (g/dL; mean ± SD) | 6.6 ± 0.5 | 6.7 ± 0.5 | 6.3 ± 0.4 | 0.10 |
| Albumin (g/dL; median [IQR]) | 3.7 (3.5–3.9) | 3.6 (3.5–3.7) | 3.6 (3.4–3.8) | 0.057 |
| Pre-dialysis creatinine (mg/dL; mean ± SD) | 11.1 ± 2.7 | 7.9 ± 1.7 | 9.2 ± 2.0 | < 0.001 |
| Modified creatinine index (mean ± SD) | 20.8 ± 2.1 | 19.8 ± 2.5 | 19.8 ± 2.6 | 0.10 |
| Use of ACE inhibitors or ARB (n [%]) | 73 (57.9) | 4 (44.4) | 7 (36.8) | 0.19 |
| Use of β-blocker (n [%]) | 26 (20.6) | 2 (22.2) | 5 (26.3) | 0.79 |

HFpEF, heart failure with preserved ejection fraction; hANP, human atrial natriuretic peptide; DW, dry weight; BMI, body mass index; CVD, cardiovascular disease; SBP, systolic blood pressure; DBP, diastolic blood pressure; SpO_2,_ peripheral oxygen saturation, CRP, C-reactive protein; ACE, angiotensin-converting enzyme; ARB, angiotensin receptor blocker; SD, standard deviation; IQR, interquartile range
